# Theory of arterial acceleration: implications for transcranial Doppler monitoring in patients with severe traumatic brain injury

**DOI:** 10.3389/fphys.2025.1654072

**Published:** 2025-10-30

**Authors:** Arjen Schaafsma

**Affiliations:** Department Clinical Neurophysiology, Martini Ziekenhuis, Groningen, Netherlands

**Keywords:** intracranial pressure, transcranial Doppler, cerebral perfusion pressure, arterial acceleration, arterial blood pressure, traumatic brain injury, monitoring, intracranial hemodynamics

## Abstract

The theory of arterial acceleration (AA) proposes that heart contraction is supported by a temporary and short-lasting contraction in arterial smooth muscle layers. Theoretically, it relies upon a stretch induced depolarization of smooth muscle cells at the aortic notch that spreads along the branches of the arterial tree via intercellular gap junctions. This wave of depolarization leads to a short-lasting contraction in the circularly arranged smooth muscle cells and generates a peristaltic wave from proximal to distal. In blood velocity or blood pressure recordings AA underlies the Sys1 component that becomes stronger the further it travels downstream. It adds to the Sys2 component, which is the pressure wave generated by the ejection of blood volume into the aorta that pushes forward the volume already present. This Sys2 component will follow the way of least resistance. The Sys1 component has a better tissue penetration than Sys2 and/or diastole which explains why intracranial pressure (ICP) elevation is accompanied by an increase in pulsatility index (PI). According to the author, the theory of AA leads to a better understanding of wave form morphology and, thereby, provides new perspectives for research into the detection, monitoring and treatment of ICP elevation.

## Introduction to the theory of arterial acceleration

After its introduction (Schaafsma, 2014), the theory of arterial acceleration (AA) is supported by new physiological and clinical observations (Schaafsma et al., 2024; Chan et al., 2025). Basically, the theory proposes that early during the ejection phase of the heart, the sudden increase in intraluminal pressure in the aorta triggers a depolarization wave in smooth muscle cells of the arterial tree. The arterial tree behaves as an electrically coupled syncytium because of the abundant presence of gap junctions (e.g., Haddock and Hill, 2005). This depolarization wave is thought to travel at its own speed from proximal to distal, effortlessly following the branching arterial tree up to the arterioles of all the bodies capillary systems. The depolarization evokes an intracellular calcium spike, hypothetically causing a brief contraction (e.g., Smith et al., 2020). The more distal we measure, the more powerful AA, since its effect builds upon the pressure generated in more proximal segments. This wave underlies the first peak in a systolic waveform: Sys1.

In contrast, the pressure wave generated by myocardial contraction results from the addition of blood volume into the ascending aorta pushing forward the volume already present. This wave will follow the path of least resistance. It is expected to dilute along the many branches of the arterial tree due to the great increase in gross cross-sectional area. This wave underlies the second peak in a systolic waveform: Sys2.

Taken together, the systolic waveform is a wave in transition: Sys2 exclusive in the aortic notch, biphasic in cephalic arteries but transforming to Sys1 exclusive in the distal arteries of the extremities. When we palpate the pulse at the radial artery, we feel heart frequency but not heart contraction: under most circumstances the systolic wave in distal arteries is entirely generated by AA.

At this point it is important to underline the fundamental differences between pressure and flow (velocity) recordings. Pressure is the driving force to flow. It is the resultant of blood volume that has been forced into the elastic capacitance formed by the aorta and its major branches (Windkessel model by Frank, (1990). Pressure is ill-maintained because there is a constant leakage of blood via the distant arteries: the volume needs to be restored by the inflow of a comparable amount of blood volume through the regular pumping of the heart.

Flow is a direct consequence of the pressure gradient: it may change instantaneously, for instance during cross-clamping of a carotid artery or, within a single beat, in a patient with subclavian steal that has a reverberating flow in the ipsilateral vertebral artery (e.g., Horrow and Stassi, 2001). In a way, flow aims to equalize the pressure difference. It can only be maintained when the pressure gradient is re-stored by balancing outflow with an equal amount of inflow.

Furthermore, it should be emphasized that pressure is defined at single locations in the arterial tree, but that flow is defined by the gradient between two locations. The fact that flow runs from proximal to distal proves that ABP should not be over-simplified as one single pressure defining the whole arterial tree (or Windkessel) but that ABP differs from one location to another. In addition, the theory of AA implies that a pressure gradient between locations (and thus the flow) is not constant over the full duration of a heart cycle: the Sys1 component gains in strength towards periphery, whereas the Sys2 component fades away. At the beginning of every heartbeat, AA squeezes out a tiny bit of volume into all the bodies capillary systems.

In this paper the author explores how the theory of AA better explains wave form morphology in TCD recordings of the middle cerebral artery (MCA) during ICP elevation. For instance, what does it mean for the pulsatility index (PI; e.g., Bellner et al., 2004). What does it mean for the detection and monitoring of ICP elevation in traumatic brain injury (TBI)? What does it imply for future therapeutic strategies?

## Connecting the theory of AA with current concepts on ICP elevation in TBI

To connect with current understanding of the patho-physiology of elevated ICP in patients with TBI a limited literature search was performed in Pubmed (https://pubmed.ncbi.nlm.nih.gov): including all studies dealing with ‘ICP monitoring’ (8,514 publications), in ‘human subjects’ (6,961 publications), with free full text availability (1,418 publications) appearing in 2015 or later (917 publications) based upon a ‘systematic review’ (24 publications).

In addition, publications were searched dealing with the ‘treatment of intracranial pressure elevation’ (27,855 publications) in ‘human subjects’ (23,685 publications), appeared in 2015 or later (8,308 publications) with free full text availability (3,117 publications) based upon a ‘systematic review’ (113 publications) in ‘traumatic brain injury’ (22 publications).

Both search methods led to 21 duplicates so that eventually a total of 25 publications was taken to reflect current understanding about the detection and treatment of ICP elevation in TBI. A list of all publications considered is added as supplement to this paper.

## Implications of the theory of AA for waveform morphology during ICP elevation

The systolic phase in blood velocity or blood pressure recordings from especially cephalic arteries is often shown to have two peaks. Standard theory explains this biphasic appearance of systole from wave reflection (e.g., Nichols et al., 2008). According to the Windkessel model of Frank, (1990) the aorta can be seen as an elastic capacitance capable of temporarily storing part of the stroke volume ejected by the heart at the cost of an increase in pressure that reliefs itself by the outflow during diastole. The sudden rise in pressure during systole spreads along the arterial tree but becomes reflected by distant points of high resistance. This causes a relative increase in pressure during the second part of the systolic wave, usually referred to as augmentation or, in the context of this paper: Sys2. In blood velocity recordings wave reflection causes a temporary dip in systolic velocity separating Sys1 from Sys2.

The debate that arteries might not be as passive to the pressure wave from the heart as generally accepted was re-initiated by Schaafsma (2014): the theory of AA postulates that Sys1 is the consequence of an active but short-lasting contraction within the smooth muscle layers of the arterial tree and adds to the pressure wave generated by the ejection of stroke volume into the limited capacitance of the aorta. Somewhat like the far end of a whip, pressure during Sys1 builds upon more proximal pressure and its contribution relative to Sys2 becomes larger with the decreasing diameter of arteries and arterioles. The further downstream, the higher the penetration force of Sys1 compared to Sys2. This can easily be demonstrated by recording the blood velocity in the radial artery during inflation of a pressure cuff placed around the hand. At cuff pressures up to 300 mmHg a Sys1 “systolic spike” remains present whereas the velocity during the later phase of systole or during diastole is completely abolished (Schaafsma, 2014).

**FIGURE 1:**
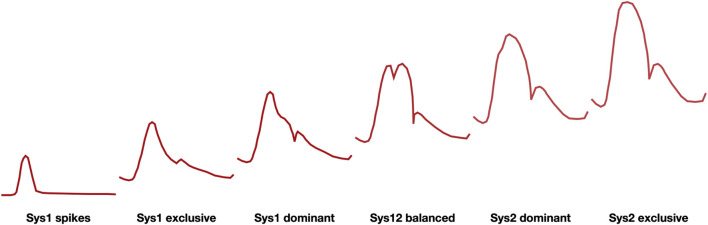
Family of waveforms for blood velocity signals in the MCA (schematic representation) ranging from systolic spikes at the far left, associated with high ICP and brain death, to sys2 exclusive at the far right, associated with hyperperfusion syndrome. Physiologically occurring wave forms are in the center.

In the MCA blood velocity recordings normally vary between Sys1 dominant, Sys12 balanced or Sys2 dominant (Figure 1) but the signal becomes increasingly Sys1 exclusive with ICP elevation. At high ICP Sys1 systolic spikes prevail and are in some countries accepted as indication of brain death (Monteiro et al., 2006).

The PI, defined by the difference between maximal and minimal blood velocity divided by the mean blood velocity will become increasingly high when the signal becomes Sys1 dominant and mean and diastolic blood velocities approach zero.

For the interpretation of MCA blood velocity, it is important that wave morphology changes with aging: from Sys1 dominant in the young the signal gradually turns into Sys12 balanced around the age of 70, reaching Sys2 dominance when over 80 years old (Schaafsma, 2018; Schaafsma et al., 2024). The interpretation of this gradual change is that with time arteries lose their elastance, become dilated and stiffer because their compliance is now determined by collagen instead of elastin fibers. That the Sys1 component becomes less prominent suggests that in the elderly the contribution of AA is less than in the young and, consequently, that therapies aimed at changing its contribution to hemodynamics may possibly be less effective in the older population.

The rapid interpretation of MCA blood velocity signal has been improved by the introduction of Z-scores (Schaafsma, 2018). Z-scores indicate how many standard deviations a given measurement deviates from that expected for normal controls at that age. By plotting Z-scores in a radar plot, MCA blood velocity signals are transformed for rapid interpretation and follow up.

**FIGURE 2:**
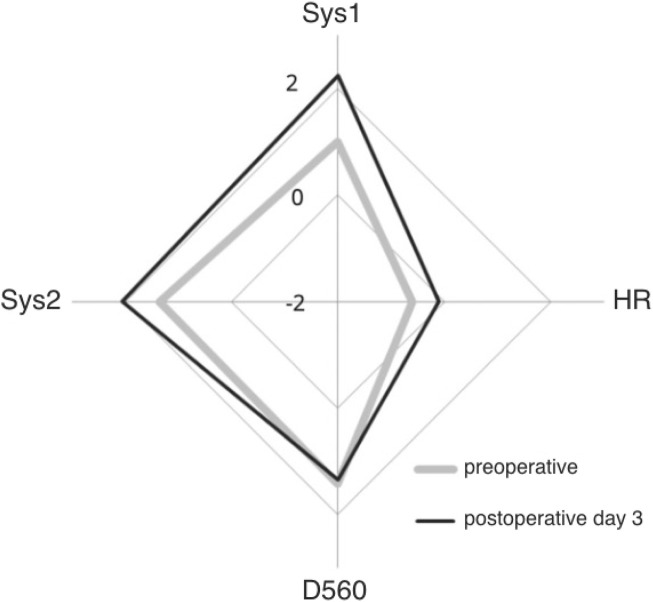
Pooled Z-scores of MCA blood velocity in over 147 patients who underwent uncomplicated ipsilateral carotid endarterectomy. Preoperative Z-scores in grey, postoperative Z-scores in black: after CEA Sys1, Sys2 and heart rate (HR) increase but D560 remains the same. Origin is minus 2 and outer diamond plus 2 standard deviations from expected for age (courtesy Edizioni Minerva Medica; Schaafsma et al., 2021).

For instance, Figure 2 shows pooled results in a group of patients who underwent carotid endarterectomy (Schaafsma et al., 2021).

## Implications of the theory of AA for future studies on the detection of elevated ICP

In literature on ICP elevation it is customary to consider cerebral perfusion pressure (CPP; e.g., Pelah et al., 2025). CPP is considered the driving force behind cerebral tissue perfusion. Diffusely elevated ICP decreases CPP since it reduces the pressure gradient between in- and outflow. The accurate determination of ICP has become a diagnostic goal on its own: it completes the formula CPP = ABP–ICP and ABP is readily available on the ICU. Note that, according to the theory of AA CPP does not have to be constant within the reach of a single heart cycle: at elevated ICP CPP is higher for Sys1 than for Sys2 and diastole, since Sys1 is more resistant to ICP elevation.

The gold standard for measuring ICP is an intraventricular catheter connected to a pressure recording device. An intraparenchymal probe may form an acceptable alternative (Zacchetti et al., 2015). In selected cases the addition of an infratentorial catheter may help to detect possible pressure gradients over the tentorium (Won et al., 2022). Severe TBI may lead to focal pressure gradients (between hemispheres or transtentorial) causing displacement or herniation of brain tissue and, thereby, focal ischemia.

In clinical practice there is a natural hesitance to perform the invasive procedure required for catheter placement unless dictated by the severity of the clinical condition. In this context, methods for non-invasive detection of ICP elevation can support clinical decision making (e.g., Bellner et al., 2004).

Nevertheless, meta-analyses have so far been unable to prove an effect of ICP monitoring on outcome or mortality (Forsyth et al., 2010; Han et al., 2016; Han et al., 2022). Unsupervised machine learning of multimodal data from the ICU has only to a limited amount shown relevance of ICP monitoring in relation to functional outcome (Tas et al., 2025). Fernando et al. (2019) have reviewed the sensitivity and specificity of non-invasive assessment of ICP elevation. They concluded that physical signs as pupillary dilatation, CT-criteria and measurement of Optic Nerve Sheet Diameter (ONSD) detected ICP elevation with only limited sensitivity.

Since therapeutic approaches solely aimed at reducing ICP still lack clinical proof, the question has arisen whether ICP reduction on its own is a sufficiently legitimate therapeutic goal (Pelah et al., 2025). Would it not be better to monitor its effect on intracranial hemodynamics more directly?

Provided a patient has sufficiently permissive transtemporal acoustic windows, monitoring of the MCA with transcranial Doppler (TCD) is a more direct way to observe the effectiveness of brain perfusion for at least 2/3rd of the upper brain volume. Intermittent TCD investigations of 1–2 min duration, e.g., starting at 1 to 2-hourly intervals and adjusting this frequency in line with clinical findings and/or interventions, is probably more practical than continuous monitoring. The latter requires mounting a head frame which, depending on the level of sedation, often leads to discomfort, unrest and elevated blood pressures: highly undesirable when recovering from TBI. For example, other clinical studies showing differences in behavior of Sys1 versus Sys2 with aging (Schaafsma et al., 2024), in sepsis (de Goede et al., 2017) and after carotid endarterectomy (Schaafsma et al., 2021) were based upon brief instead of prolonged periods of MCA monitoring; the latter two on repetitive monitoring.

The theory of AA predicts that the PI will increase when ICP rises. On its own, however, the PI is a ‘noisy’ parameter, since it is ill-defined (Schaafsma, 2014) and depends on age, on heart rate and on ABP (Schaafsma, 2018). Based upon 4 studies, Fernando et al. (2019) saw no role for Pulsatility Index (PI) measurement with TCD because of a low Area Under Receiving Operator Curve (AUROC) varying between 0.55 and 0.72 for the detection of an ICP of 20 mmHg or higher. Combining TCD with ABP measurements, however, increased the AUROC to 0.85 (95% Confidence Interval (CI) 0.79–0.91). Brasil et al. (2024) also found that the sensitivity of the PI can be increased by comparing TCD-PI with ABP-PI: in TBI patients calculating the pulsatile apparent resistance for Sys1 and end diastolic velocity resulted in an AUROC of 0.77 (95% CI 0.61–0.85) for the detection of an ICP of 25 mmHg or higher and up to 0. 88 (95% CI 0.72–1.00) in TBI patients without neurosurgical procedure.

The theory of AA better explains why in elevated ICP the waveform of MCA blood velocity changes from Sys1 dominant to Sys1 exclusive and, ultimately, systolic spikes. The author is convinced that a better interpretation of the systolic wave form will lead to better clinical decision making. For this, however, more experience with systolic waveform analysis in TBI is required.

## Implications of the theory of AA for future research on the treatment of TBI

Treatment of severe traumatic brain injury (TBI) follows the Monro-Kellie doctrine (Cushing, 1926). Any space requiring process, such as neoplasm, hemorrhage, oedema, venous obstruction, CSF resorption problem, etc. forms a challenge to the limited physiological capacitance of the skull. Normal intracranial components, namely, brain tissue, blood and cerebrospinal fluid, can only absorb part of the pathological demand for space and, when limits are exceeded, ICP will rise.

As argued before, a gradual increase in ICP makes the MCA blood velocity signal increasingly Sys1 dominant, ultimately turning into systolic spikes (Figure 1). Once aware of this systematic change, intensivists are expected to better time ancillary investigations (such as CT) and/or therapeutic interventions (such as, for instance, a change in respiratory parameters, drug administration or neurosurgical intervention). Though there is a large inter-individual variation in MCA blood velocity, the intra-individual variation of repeated or continuous measurements is significantly less and more closely related to physiological and patho-physiological changes (e.g., Bögli et al., 2024). It brings us to the question how to translate signs and symptoms in individual patients with TBI to an optimized therapeutic approach.

From a physiological point of view, any possible therapeutic intervention falls into one of four categories: 1. Optimize blood glucose and oxygen, 2. Reduce metabolic demand by reducing neuronal activity, 3. Promote blood supply to as much viable brain tissue as possible and 4. Taper excessive inflammation caused by structural damage.

1. Optimization of blood glucose and oxygen, being the only two constituents of cerebral metabolism, is core business on the ICU (Hays et al., 2022; Chang et al., 2025). The washout of CO2, however, is not always closely monitored. Since pCO2 is almost linearly related to local cerebral blood flow, it is significant: systemic hypocapnia ‘fools’ neurovascular coupling and leads to local cerebral ischemia, whereas systemic hypercapnia results in a rise in ABP with cerebral hyperemia risking more brain oedema and ICP elevation (Chan et al., 2025). Hyperventilation makes the MCA blood velocity more Sys1 dominant, whereas CO2 retention makes the signal more Sys2 dominant (Schaafsma, 2014; Chan et al., 2025). Apart from optimizing oxygenation and blood glucose, pCO2 shall always be kept within the normal physiological range.

2. The reduction of metabolic demand is usually accomplished by limited sedation since a more profound medical coma can have deleterious side-effects on ABP and cerebral autoregulation. The occurrence of clinical or subclinical epileptic seizures should be detected and treated early in its development since epilepsy is an important reason for an increased metabolic demand. Other strategies for decreasing metabolic activity such as cooling have so far not led to a more positive outcome (Martyniuk et al., 2024).

3. Promoting blood supply to as much viable brain tissue as possible can theoretically be achieved by increasing ABP, by increasing cardiac output, by increasing AA and by increasing heart rate on one side and by promoting venous outflow on the other. Common strategies aim to reduce ICP by improving venous outflow (head up) and/or CSF resorption/drainage. Improving CPP by the administration of norepinephrine in TBI patients with hypotension has not unequivocally resulted in a better outcome (Lloyd-Donald et al., 2020). Treatments aimed at reducing ICP by, for instance, a continuous infusion of hypertonic saline have failed to show an improvement in outcome (Bernhardt et al., 2024). Craniotomy and craniectomy are last resort neurosurgical interventions that although potentially lifesaving, because of a negative selection bias, are not necessarily associated with less severe neurological sequelae (Sahuquillo and Dennis, 2019).

From the perspective of AA, a Sys1 dominant or Sys1 exclusive signal indicates last resort tissue perfusion. Care should be taken not to deteriorate the Sys1 pulse, for instance, by avoiding certain anti-hypertensives, such as ACE inhibitors and calcium inhibitors (e.g., Laurent et al., 2022). Therapies may even aim at the promotion of Sys1 penetration into cerebral tissue (e.g., by intravenous administration of dopamine; personal observation). At present, however, the differential effect of pharmacological agents upon either Sys1 or Sys2 is not well understood and requires further study.

4. Reduction of possibly too aggressive local inflammation caused by structural damage has so far not been successful. Cooling in TBI, that can also be seen as tapering inflammation, has no proven effect on functional outcome or mortality (Martyniuk et al., 2024). Application of corticosteroids has even been shown detrimental in TBI (Wang et al., 2025). Local inflammation is likely to disturb metabolic coupling. It is expected to be accompanied by high local blood velocities over the MCA indicating hyper flow, comparable to the high blood velocities found in sepsis (de Goede et al., 2017).

In summary, this paper explores how a better understanding of the physiological factors contributing to the systolic waveform of MCA blood velocity may allow a better assessment of intracranial hemodynamics in patients with TBI. In future, TCD may become increasingly helpful for the monitoring and treatment of patients with severe TBI. In particular, the notion that AA maintains a rudimentary tissue perfusion despite elevated ICP and, therefore, should be preserved and protected has potential therapeutic consequences. Intermittent TCD-monitoring of the MCA provides direct information on regional brain perfusion, covering at least 2/3rd of the upper brain when applied bilaterally. Any monitoring of ICP or intracranial hemodynamics should ultimately prove its beneficial effect on outcome and/or mortality. For now, its introduction in a treatment protocol should balance the gain of providing additional information to negative side-effects for the patient’s wellbeing.

## Data Availability

The original contributions presented in the study are included in the article/Supplementary Material, further inquiries can be directed to the corresponding author.
